# Angle-Selective Photonics for Smart Subambient Radiative Cooling

**DOI:** 10.1007/s40820-025-01698-0

**Published:** 2025-03-10

**Authors:** Fan Liu, Qichong Zhang

**Affiliations:** Key Laboratory of Multifunctional Nanomaterials and Smart Systems, Suzhou Institute of Nano-Tech and Nano-Bionics, Chinese Academy of Sciences, Suzhou, 215123 People’s Republic of China

**Keywords:** Radiative cooling, Vertical surface, Angular asymmetry

## Abstract

During the daytime, conventional radiative coolers disregard the directionality of thermal radiation, thereby overlooking the upward radiation from the ground. This upward radiation enhances the outward thermal radiation, leading to a substantial reduction in the subambient daytime radiative cooling performance. Conversely, radiative coolers featuring angular asymmetry and spectral selectivity effectively resolve the problem of thermal radiation directionality, successfully evading the interference caused by the ground-generated thermal radiation. This cooler overcomes the limitations posed by the angle of incident light, making it suitable for subambient daytime radiative cooling of vertical surfaces. Furthermore, by adjusting the structure of the cooler, the angular range of thermal radiation can be modulated, enabling the application of radiative cooling technology for intelligent temperature regulation of various inclined surfaces encountered in daily life. This innovative work makes a significant contribution to the development of subambient smart thermal interaction systems and opens up new possibilities for the practical application of radiative cooling technology.

## Highlights

- Subambient daytime radiative cooling of vertical surfaces is achieved by a sawtooth grating with a period significantly greater than the thermal wavelength.
- Adjusting the grating period and aspect ratio allows the cooler to be adapted to various inclined surfaces.

## Graphical abstract

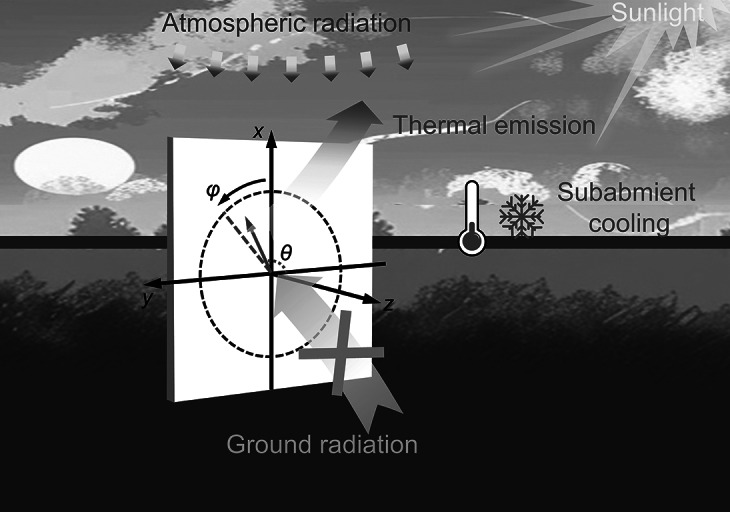

## Introduction

The progress of human society hinges on the utilization of energy. However, the consumption of non-renewable energy has led to an increase in greenhouse gas emissions [1]. The ensuing issue of global warming has compelled a sharp rise in demand for cooling solutions, imposing a huge burden on the environment. In light of current development needs, cooling technology is no longer confined to temperature regulation. It is evolving toward achieving high-performance, integrating advanced intelligence, and giving priority to environmental sustainability. In this context, radiative cooling technology has emerged as a promising passive cooling strategy [1]. This technology harnesses a natural phenomenon of energy transfer called photon heat flow to dissipate energy and entropy into outer space. By doing so, it achieves cooling effects without the need for additional energy input [2]. This revolutionary approach is expected to facilitate the integration and development of various disciplines, including materials science, energy development, and intelligent systems. It holds the potential to usher in a new era of zero-emission cooling solutions worldwide.

Manipulating materials and microstructures allows for control of light across a wide spectral range and is thus considered a highly effective method for modulating subambient daytime radiative cooling. The current research primarily focuses on the direct impact of solar radiation on the surfaces of radiators that are oriented toward the sky, facilitating the outward emission of infrared heat. Based on this principle, various photonic metasurfaces [3], such as periodic arrays and multilayer film structures, have been designed to maximize thermal emission. However, a significant gap in the current research is the neglect of upward radiation from the ground, especially during daylight hours when surfaces quickly heat up due to solar exposure, significantly enhancing outward thermal radiation. Additionally, fluctuating weather conditions and mobile targets create dynamic temperature fluctuations, posing challenges for achieving high-efficiency subambient daytime radiative cooling. In essence, the current limitation of radiative cooling technologies in subambient environments is the lack of "vectorization," which requires not only consideration of cooling performance but also directional control. To break this directional deadlock, previous thermal radiators with photonic metasurfaces exhibited central symmetry, resulting in angularly symmetric thermal radiation. Zhou et al. have introduced a novel approach by utilizing periodically tilted wedge-shaped cavities [4], which introduces "direction" to microstructures and breaks the symmetry of radiative coolers. This innovative technique successfully manipulates the direction of thermal radiation, paving the way for more advanced and efficient radiative cooling solutions.

Imagine a scenario where the direction of thermal radiation could be extended to the normal direction of vertical surfaces, which would have a profoundly positive impact on energy-saving in thermal management for building facades and vehicle sides. Radiative cooling technology on these vertical surfaces not only aims to counteract the negative effects of ground thermal radiation but also strives to minimize the absorption of solar energy to the utmost degree. The latest study, published in Science by Xie et al., has successfully expanded the scope of subambient daytime radiative cooling from horizontal to vertical surfaces [5], completely breaking through the constraints of light angles and achieving impressive progress.

**Fig. 1:**
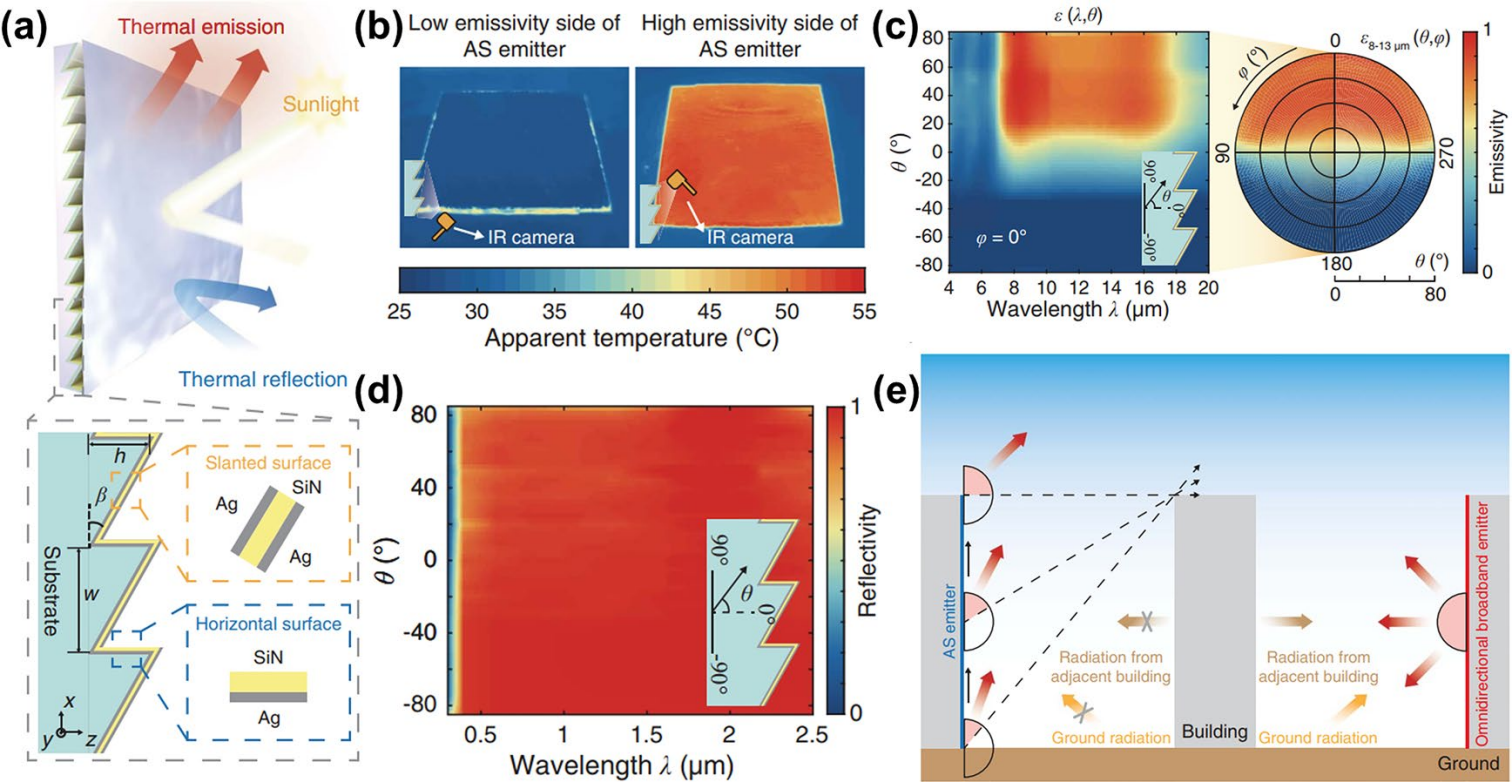
**a** Schematic of the radiative cooler consisting of a sawtooth grating, **b** thermography images of the radiative cooler taken from opposing directions, **c** spectral angular emissivity of the radiative cooler, **d** simulated spectral solar reflection of the radiative cooler under different incident angles, and **e** schematic of the impact of adjacent buildings on the gradient the radiative cooler and omnidirectional broadband emitter

Xie et al. have the foresight to employ a sawtooth grating with periodicity to construct a radiative cooler that features both angular asymmetry and spectral selectivity (Fig. 1a). In their innovative structural design, they deliberately altered the planar mirror symmetry of the sawtooth structure, thereby achieving angularly asymmetric thermal emission (Fig. 1b). By combining the directional polarization resonance of the horizontal surface of the sawtooth grating with a single layer of silver and silicon nitride, they were able to achieve a spectrally selective thermal emission at approximately 11 μm (Fig. 1c).

The synergistic effect achieved through the interplay of the slanted double-layer silver, the single-layer silicon nitride, and the overlaying nanoporous polyethylene film, not only effectively counteracts ground thermal radiation but also ensures strong reflection across the entire solar wavelength range (Fig. 1d), satisfying the stringent reflectivity requirements of the solar spectrum.

Once the directional constraints of the subambient daytime radiative cooling system are broken, intelligent thermal interaction systems will unlock new possibilities for thermal regulation. The radiative cooler invented by Xie et al. utilizes a meticulously designed grating period and aspect ratio to effectively adjust the angular range of thermal radiation, making it applicable for intelligent cooling of various inclined surfaces in daily life. For instance, when applied to building walls, this radiative cooler can fully leverage its advantages for efficient cooling on vertical surfaces (Fig. 1e). The dynamic cooling effect resulting from this angular asymmetry significantly surpasses that of commercial white paints and other radiative coolers, highlighting its exceptional performance in intelligent thermal interaction for subambient cooling. Furthermore, the radiative cooler with vectorized optical properties has effectively addressed the limitations of directionality, paving the way for its application in the domain of irregular wearable textiles. Regardless of the angle of incident light, this cooler could demonstrate exceptional radiative cooling performance, representing a significant breakthrough in the application of radiative cooling technology.

This research holds significant implications for various fields. Theoretically, it presents innovative design ideas and methodologies that revolutionize the field of subambient daytime radiative cooling. It challenges conventional design concepts in radiative heat transfer systems, fostering further advancements in related theories and paving the way for new possibilities. From a practical standpoint, this study offers feasible solutions to cooling demands in diverse fields. It broadens the application scope of radiative cooling technology, accommodating various industries such as architecture, transportation, and textiles. This technology holds immense potential to play a pivotal role in the future energy-efficient building, enhancing vehicle thermal management, and delivering comfortable textiles. However, to realize the vision of stable, scalable, and cost-effective intelligent thermal interaction, further advancements in angularly asymmetric radiative coolers are essential. For example, the necessary structural optimizations and the introduction of advanced materials will aid in enhancing the mechanical properties and self-cleaning capabilities of radiative coolers, thereby extending their lifespan in outdoor environments. Moreover, to meet the coloring requirements of radiative coolers, the spacing of periodic gratings can be adjusted to manipulate the photonic structures, thereby producing vibrant structural colors. Additionally, further research is warranted to explore adaptive temperature regulation technologies within subambient intelligent thermal interaction systems. The combination of angularly asymmetric radiative coolers with self-switching radiative cooling materials (such as VO_2_) can intelligently modulate temperatures while serving esthetic purposes, achieving true intelligent thermal perception and interaction. It is anticipated that this technology will continue to evolve and improve, significantly contributing to mitigating global warming and reducing energy consumption. Undoubtedly, this will open new chapters in the practical applications of radiative cooling technology, ushering in a new era of intelligent thermal management.
